# Durable Response to Combined Nivolumab, Lenvatinib, and Radiation Therapy Against Oligometastatic Hepatocellular Carcinoma

**DOI:** 10.7759/cureus.73434

**Published:** 2024-11-11

**Authors:** Sewoon Chun, Erin M Coyne, Jeffrey Meyer, Won Jin Ho

**Affiliations:** 1 Public Health Sciences, University of Maryland, College Park, USA; 2 Oncology, Johns Hopkins University School of Medicine, Baltimore, USA; 3 Radiation Oncology and Molecular Radiation Sciences, Johns Hopkins University School of Medicine, Baltimore, USA

**Keywords:** checkpoint inhibitor therapy, combination cancer immunotherapy, hepatocellular carcinoma (hcc), lenvatinib, oligometastasis, opdivo nivolumab, radiotherapy (rt)

## Abstract

Hepatocellular carcinoma (HCC) is the most common type of liver cancer and is associated with major risk factors such as hepatitis B virus (HBV), hepatitis C virus (HCV), alcoholic fatty liver disease, and metabolic dysfunction-associated steatotic liver diseases. Despite the recent progress in systemic treatment regimens involving immunotherapies and targeted therapeutics, advanced HCC remains difficult to control. Moreover, with several treatment modalities currently available for HCC such as radiation therapy, transarterial chemoembolization (TACE), checkpoint immunotherapies, and multi-tyrosine kinase inhibitors, it is unclear what combination yields the greatest treatment efficacy and durability. Here, we present the case of a male patient in his 60s with HCV-associated cirrhosis diagnosed with HCC with a metastatic lesion to the T9 spine. Treatment with nivolumab and subsequently lenvatinib in addition was complicated by adverse effects including hand rash and kidney injury. Systemic therapy was stopped, and consolidative stereotactic body radiation therapy (SBRT) was delivered to the sites of the disease. The combination proved to be highly durable without any evidence of progression for over three years despite having stopped all therapy. All toxicities have resolved since, and the patient remains very active. This case demonstrates the feasibility of combining therapeutic modalities to achieve exceptional disease control in the setting of oligometastatic disease.

## Introduction

Hepatocellular carcinoma (HCC), associated with various liver injury diseases such as hepatitis viruses, alcohol use, and metabolic-associated fatty liver disease [1], is the most common form of liver cancer. HCC is a highly lethal cancer type placed among the top five causes of cancer-related deaths worldwide [2]. Its tendency to remain asymptomatic until late stages with resistance to conventional cytotoxic chemotherapies without many other options for systemic therapies has contributed to poor outcomes, with advanced HCC associated with a five-year survival rate of 5-14% [3]. Within the past decade, however, research brought forward multiple breakthroughs in the development of immunotherapies and targeted therapies. An FDA-approved first-line therapy for advanced HCC is a combination of atezolizumab and bevacizumab, which employs an immune checkpoint programmed death-ligand 1 (PD-L1) inhibitor and an inhibitor of vascular endothelial growth factor (VEGF), respectively. Atezolizumab plus bevacizumab has shown to be superior against previous generations of targeted therapies such as sorafenib, a multikinase inhibitor [4]. However, as more effective systemic therapies become available as viable treatment options, it remains unclear whether to incorporate other forms of therapy such as dose-escalated radiation therapy (RT) including stereotactic body radiation therapy (SBRT) and transarterial chemoembolization (TACE) to potentially yield therapeutic synergy and/or extended durability. For example, in the setting of oligometastatic HCC, the current literature does not prescribe the best combination of systemic therapy and other treatment modalities. Here, we present the case of a male patient in his 60s diagnosed with hepatitis C virus (HCV) and oligometastatic HCC who was treated with systemic therapies followed by SBRT, showing an especially durable response.

## Case presentation

**Figure 1:**
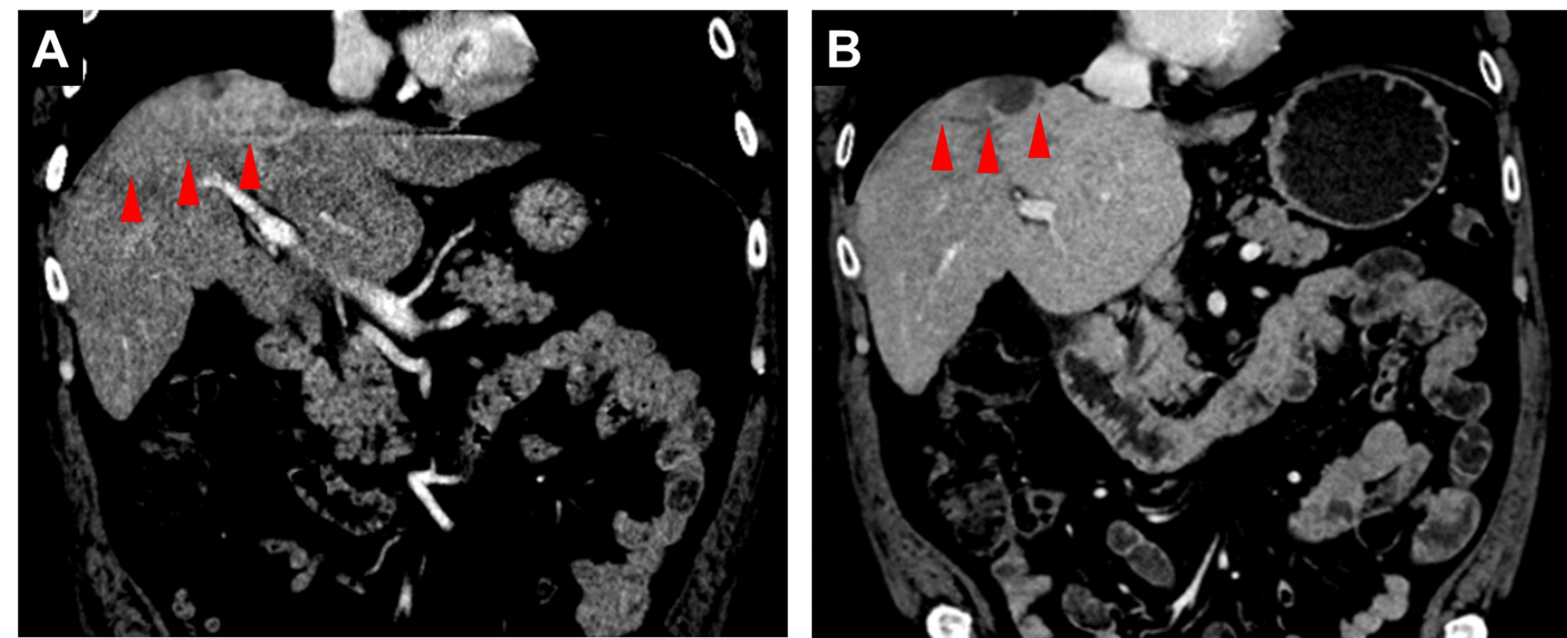
Restaging contrasted CT scans (A) Before treatment, the CT scan shows a multilobulated hepatic mass on the right lobe of the liver measuring 5.4×7.5×4 cm in size (red arrows). (B) After systemic therapy and radiation therapy, scans reduced the disease burden.

A man in his 60s presented with a solitary hepatic mass in the setting of cirrhosis related to untreated HCV cirrhosis. The mass was consistent with HCC based on imaging features, and the Child-Pugh score was A5. The patient was highly active upon presentation but was found to have a bleeding gastric ulcer, during the diagnosis of which a new legion on his liver was found. Biochemical tests demonstrated a markedly high alpha-fetoprotein (AFP) level, the most established blood-based biomarker for HCC, at 10,275 ng/ml, suggestive of an aggressive phenotype. MRI evaluation indicated mild to moderate hepatic steatosis and liver nodularity consistent with HCV-associated cirrhosis and an arterially enhancing hepatic mass with venous washout characteristic of HCC measuring 5.4×7.5×4 cm (Figure 1A). A staging CT scan revealed a destructive area involving the left side of the T9 vertebral body, indicating metastasis and establishing the stage as Barcelona Clinic Liver Cancer stage C (BCLC-C). The patient did not endorse any back pain or palpable tenderness along the spine.

**Figure 2:**
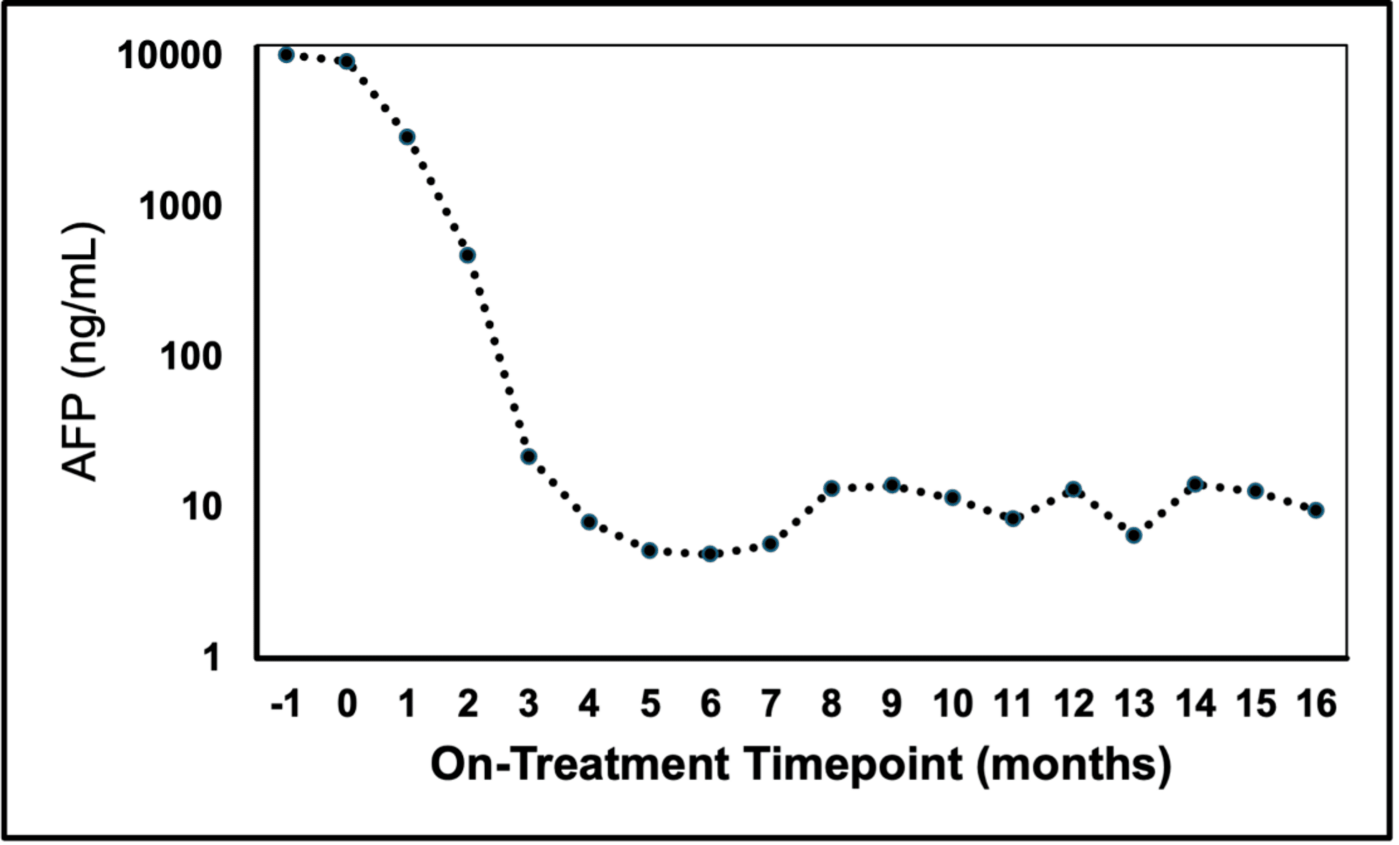
AFP throughout the treatment course AFP levels were tested periodically throughout the course of the treatment and subsequent follow-up visits. AFP levels (ng/ml) are shown on a logarithmic scale. With the administration of nivolumab and lenvatinib, AFP levels dropped significantly with stabilization. AFP: alpha-fetoprotein

The diagnosis of metastatic HCC precluded curative options such as surgery, microwave ablation, and liver transplantation. Though bevacizumab plus atezolizumab was an approved first-line therapy for advanced HCC, due to the patient's recent major gastrointestinal bleeding event, an inhibitor of programmed cell death protein 1 (PD-1) nivolumab, which was an alternative first-line therapy at the time, was promptly started at 480 mg per dose on a monthly basis. Just after a cycle of nivolumab, lenvatinib was also added to the regimen at a lower dose of 8 mg daily. Within two months of starting lenvatinib, the patient experienced dryness and discomfort in his hands that gradually increased in severity. After five cycles of nivolumab, the patient also developed an increase in creatinine levels as high as 1.91 mg/dl, clinically attributed to immune-related renal toxicity. In response to these adverse effects, 10% urea cream and a short empirical course of prednisone (0.5 mg/kg/day) were administered, respectively, after which there was a resolution of his skin discomfort and kidney injury. Further nivolumab was held. No other side effects or symptoms such as fatigue, fevers, nausea, diarrhea, other rashes, dyspnea, or edema were noted. Throughout the treatment course, AFP levels markedly dropped from 10,275 ng/ml at baseline to a nadir of 4.9 ng/ml by the end of systemic therapy (Figure 2). Given the potential for exacerbating toxicities, strategies to further consolidate the observed responses were pursued. The patient subsequently underwent SBRT to the liver mass (2700 cGy in three fractions) and the T9 lesion (2000 cGy to the evident disease in T9 and 1400 cGy to the remainder of the vertebral body, in one fraction, using a simultaneous integrated boost approach) [5]. Upon completion of radiation, the resumption of systemic therapies was deferred indefinitely. Notably, AFP levels remained low after SBRT, fluctuating between 5.7 and 14.3 ng/ml with the latest measurement more than three years after treatment cessation at 9.6 ng/ml. The patient continued to demonstrate no radiographic evidence of active disease (Figure 1B), with expected post-radiation changes noted in the liver, and no clear progression of the irradiated vertebral level [6]. Throughout the course of systemic and radiation treatment as well as after stopping all therapy, the patient's performance status remained robust, staying active and being able to carry out pre-disease tasks such as working full-time.

## Discussion

Significance of the observed response

HCC, through the BCLC staging system, is classified as advanced when there is major vascular invasion and/or extrahepatic spread, in which case the disease is no longer considered curable. Although the current literature varies widely on the expected survival rate of metastatic HCC, it is overall considered to be associated with poor clinical outcomes. Historically, metastatic HCC has been associated with a five-year survival rate of 2%, and more contemporary data describes less than 20% five-year survival [7,8]. A 2020 study using the Surveillance, Epidemiology, and End Results (SEER) database found that the median overall survival of HCC patients after diagnosis, depending on the etiology, ranges from 6.1 months to 10.3 months [7]. Our presented case was characterized by oligometastatic disease to the spine. HCC with spinal metastasis is associated with poor prognosis with a median overall survival of approximately 6-7 months even with RT [9,10]. Compared to these statistics, the presented case, especially in the setting of complete treatment cessation for over two years, demonstrates exceptional durability of response.

Rationale for the specific VEGF-receptor/immuno-oncology (VEGF-R/IO) combination

At the time of this report, the best-approved combination of VEGF-R-targeted therapy and checkpoint immunotherapy is bevacizumab plus atezolizumab. The IMBrave150 trial demonstrated that this combination therapy is superior compared to sorafenib with a median overall survival rate of 19.2 months versus 13.4 months [3]. Other similar combinations of therapies have been recently considered such as lenvatinib and pembrolizumab in the LEAP-002 study [11]. This phase 3 randomized double-blind study compared lenvatinib and pembrolizumab versus lenvatinib alone, resulting in the longest median overall survival of 21.2 months versus 19.0 months, respectively [11]. Although the study did not meet the pre-specified statistical criteria, together with the IMbrave150 trial, it demonstrated that combining VEGF-R-targeted therapy with checkpoint inhibition is a viable therapeutic strategy. In this case, due to the initial presentation of a bleeding ulcer, we opted to initiate treatment immediately with a PD-1 checkpoint inhibitor and follow a month later with targeted therapy. The choice to add lenvatinib was also advantageous given the ability to modify the dosing and shorter half-life compared to bevacizumab. Indeed, there were no significant bleeding events throughout the treatment course.

Evidence for the addition of RT to immunotherapy

The current guidelines from the American Association for the Study of Liver Diseases for the treatment of HCC do not prescribe SBRT as a first-line treatment option for any stage of HCC and do not explicitly consider SBRT in advanced-stage HCC [12]. However, there is growing evidence that RT may cooperate with immunotherapy to augment immune responses as well as the durability of treatment, which has been reviewed separately in-depth [13]. On the one hand, RT may serve to stimulate the tumor microenvironment to make cancer cells better recognized by the immune system; on the other hand, RT after immunotherapy may serve as a useful consolidation measure to effectively extend disease control. Radiation may prime the immune response to tumor-associated antigens and synergize with immunotherapy treatments [14]. In a retrospective observational study with 76 patients diagnosed with advanced-stage HCC, a comparison was made between immune checkpoint inhibitors and antiangiogenic therapy with or without concurrent RT [15]. When comparing the 33 patients in the RT group versus the 43 patients in the non-RT group, the RT group demonstrated much better outcomes, having a median progression-free survival of 8.3 months and an objective response rate (ORR) of 75.9% compared to the non-RT group of 4.2 months and 24.1%, respectively [15]. The study concludes by stating that this triple therapy demonstrates improved survival outcomes and disease control rate compared to a non-RT version of the treatment. In another retrospective study of five patients with unresectable HCC, a combination of TACE, SBRT, and anti-PD-1 therapy was used demonstrating positive outcomes. The ORR was 100%, with 40% of the patients having a complete response and the rest partial response [16]. The median progression-free survival was 14.9 months with one-year local control and overall survival rates of 100%, though it is important to note the study's small sample size [16]. In addition, RT can be used during or after immunotherapy as well to increase the durability of treatment response. In the context of extensive small cell lung cancers, consolidative RT after chemoimmunotherapy is increasingly supported [17,18]. Similarly, a study assessing the role of SBRT on oligoprogressed lesions in patients with non-small cell lung cancer treated with checkpoint inhibitors revealed favorable outcomes. The one- and two-year rates of local control of these oligoprogressed regions were 100% and 81.8%, respectively [19]. Another study involved 30 patients with advanced solid tumor cancers, with the majority of patients having either HCC or urothelial carcinomas undergoing immune checkpoint inhibitor therapy. Patients in this study underwent salvage RT after oligoprogression on immunotherapy and exhibited favorable clinical outcomes with overall survival rates of 100%, 96.3%, and 82.8% at six, 12, and 24 months, respectively [20]. Together, these studies support the utility of RT before, during, and/or after immunotherapy to enhance overall clinical efficacy. Our presented case is consistent with these lines of evidence and suggests that RT is an important modality to consider even in the setting of advanced HCC. Neither this current case nor the literature is particularly clear on what may be the most optimal sequence and dosing in combining these modalities, and more clinical trials are needed for investigation.

## Conclusions

The combination of nivolumab and lenvatinib followed by SBRT demonstrated an exceptional response in this case of metastatic HCC, maintaining no sign of active disease without any additional therapies for >3 years, far past the expected median. Though the current treatment guidelines currently do not advise on the most optimal combination of various treatment modalities available for use, our case suggests that a judicious combination of nivolumab, lenvatinib, and SBRT was responsible for a highly durable disease control in a patient who presented with a significant gastric bleeding event and subsequently suffered treatment-related toxicities. Furthermore, disease control was achieved without any significant compromise in performance status upon recovery from observed toxicities. The exceptional response from this patient leverages several breakthrough advances in treating HCC. With exciting progress in managing HCC, further exploration of which modalities to combine as well as the sequence in which they are employed is critically warranted. These efforts would be key to realizing the maximum potential of these therapeutic options.
